# Chronic Drought Decreases Anabolic and Catabolic BVOC Emissions of *Quercus pubescens* in a Mediterranean Forest

**DOI:** 10.3389/fpls.2017.00071

**Published:** 2017-02-08

**Authors:** Amélie Saunier, Elena Ormeño, Henri Wortham, Brice Temime-Roussel, Caroline Lecareux, Christophe Boissard, Catherine Fernandez

**Affiliations:** ^1^Aix Marseille Univ., Univ. Avignon, CNRS, IRD, IMBEMarseille, France; ^2^Aix Marseille Univ., CNRS, LCE, Laboratoire de Chimie de l'EnvironnementMarseille, France; ^3^Laboratoire des Sciences du Climat et de l'Environnement, LSCE/IPSL, CEA-CNRS-UVSQ, Unisersité Paris-SaclayGif sur Yvette, France

**Keywords:** amplified drought, anabolism, catabolism, BVOC, VOC oxidations

## Abstract

Biogenic volatile organic compounds (BVOC) emitted by plants can originate from both anabolism (metabolite production through anabolic processes) and catabolism (metabolite degradation by oxidative reactions). Drought can favor leaf oxidation by increasing the oxidative pressure in plant cells. Thus, under the precipitation decline predicted for the Mediterranean region, it can be expected both strong oxidation of anabolic BVOC within leaves and, as a result, enhanced catabolic BVOC emissions. Using an experimental rain exclusion device in a natural forest, we compared the seasonal course of the emissions of the main anabolic BVOC released by *Q. pubescens* (isoprene and methanol) and their catabolic products (MACR+MVK+ISOPOOH and formaldehyde, respectively) after 3 years of precipitation restriction (−30% of rain). Thus, we assume that this repetitive amplified drought promoted a chronic drought. BVOC emissions were monitored, on-line, with a PTR-ToF-MS. Amplified drought decreased all BVOC emissions rates in spring and summer by around 40–50 %, especially through stomatal closure, with no effect in autumn. Moreover, ratios between catabolic and anabolic BVOC remained unchanged with amplified drought, suggesting a relative stable oxidative pressure in *Q. pubescens* under the water stress applied. Moreover, these results suggest a quite good resilience of this species under the most severe climate change scenario in the Mediterranean region.

## Introduction

Continental biosphere contributes to global emissions of biogenic volatile organic compounds (BVOC) with an estimated emission rate of 1 PgC.yr^−1^ (Guenther et al., 1995; Harrison et al., 2013). The main BVOC emitted by plants is isoprene, with an annual emission rate of 400–600 TgC.yr^−1^ (Arneth et al., 2008) and methanol, with annual emissions ranging between 122 and 350 Tg.yr^−1^ (Tie et al., 2003; Singh et al., 2004; Jacob et al., 2005; Millet et al., 2008). Both, isoprene and methanol are considered as anabolic BVOC since they are produced through anabolic processes as defined by Oikawa and Lerdau (2013). Precursors of isoprene (DMAPP and IPP) are produced by methylerythritol-4-phosphate (MEP) and mevalonate pathway (MVA) but isoprene is synthesized through MEP pathway the in the chloroplasts (Lichtenthaler, 1999; Pazouki and Niinemets, 2016). This molecule is known to be a defense molecule against abiotic stresses, especially drought, with variable responses according to stress severity (Peñuelas and Staudt, 2010). As a whole, increases are mostly observed when water stress is moderate, (Funk et al., 2004; Pegoraro et al., 2005), and decreases when water stress is severe (Brüggemann and Schnitzler, 2002; Fortunati et al., 2008). Moreover, seasonality of isoprene emissions is well-known with the highest emission rates in summer (Goldstein et al., 1998). After its production, isoprene can be oxidized in different products including methacrolein (MACR), methyl vinyl ketone (MVK), and isoprene hydroxy hydroperoxides (ISOPOOH) (Jardine et al., 2013; Kalogridis et al., 2014). However, the effect of drought and seasonality on isoprene oxidation products has never been studied before.

Methanol emissions, such as other oxygenated BVOC with one carbon, are strongly bound to the vegetal growth (Kreuzwieser et al., 1999). Methanol is formed through pectin demethylation in plant cell walls (Fall, 2003; Oikawa and Lerdau, 2013). This compound could be considered as an indicator of growth process (Folkers et al., 2008) and, consequently, its seasonal course follows this process (Karl et al., 2003). Methanol can be degraded into formaldehyde (Kotzias et al., 1997) but its emissions originated from plants have been poorly investigated (Seco et al., 2008), and never under water stress as well as seasonality.

Oxidation reactions of isoprene and methanol with OH radicals, O_3_, and NO_x_, are well-known to occur in the atmosphere (Calogirou et al., 1999; Atkinson and Arey, 2003), but these reactions can also occur within leaves with reactive oxygen species (ROS, Oikawa and Lerdau, 2013). In this case, catabolic BVOC emissions, formed by oxidation of anabolic BVOC, will be observed (e.g., formaldehyde, isoprene oxidation products). Because of these cellular oxidative reactions, anabolic BVOC are considered as ROS scavengers, while catabolic foliar emissions have been suggested as indicators of both oxidative stress and recycling carbon (Oikawa and Lerdau, 2013). Catabolic BVOC have thereby been suggested to play a role in the tolerance of environmental stress and in plant-atmosphere interactions (Peñuelas and Staudt, 2010; Oikawa and Lerdau, 2013). Although interesting information about plant stress arises from catabolic emission analysis, there are large uncertainties associated with these emissions since (i) they can have a biogenic or anthropogenic origin (ii) they can be primary or secondary—i.e., oxidized by catabolic reactions in leaves (primary) or oxidized in the atmosphere (secondary), (iii) their measurements can be complex because of the strong affinity of some compounds to water (e.g., formaldehyde), (iv) vegetation can act as a sink (deposition) or a source of catabolic BVOC (Guenther, 2013), (v) large uncertainties also remain on how climate change might affect catabolic BVOC through the seasonal cycle, especially when drought (expected with climate change) is applied during few years, defining a chronic drought (Niinemets, 2010b; Brzostek et al., 2014). Indeed, chronic reduced rainfall have progressive and cumulative effects over time, especially on forested ecosystems dominated by long-lives species (Smith et al., 2009).

In the Mediterranean area, climatic models predict an intensification of the chronic summer drought, typical of this region, for 2100 with the most severe scenario of global change. This scenario involves a rain reduction that can, locally, reach 30% (Giorgi and Lionello, 2008; IPCC, 2013) and an extension of drought period (Polade et al., 2014). It is thereby expected a change in primary (growth) and secondary (defense) metabolisms in trees that could consequently modify the anabolic and catabolic BVOC emissions. The model selected here is the Downy Oak (*Quercus pubescens* Willd.) which is well-widespread in the northern part of the Mediterranean basin (Quézel and Médail, 2003) occupying 2 million ha (personal communication from T. Gauquelin). It also represents the major source of isoprene emissions in the Mediterranean area (Simon et al., 2005; Keenan et al., 2009) and, the impact of watering withholding has only been studied under short term periods (Rodríguez-Calcerrada et al., 2013; Genard-Zielinski et al., 2014). The impact of chronic drought on trees in terms of physiology is not well-understood but it seems that this phenomenon can decrease the trees growth (Brzostek et al., 2014) and, in terms of BVOC emissions, this impact has been poorly tackled (Lavoir et al., 2009).

Using a rain exclusion device installed in a natural Downy Oak forest, we aimed to evaluate the effect of a chronic amplified drought (restriction precipitation of 30%) applied since 2012 and repeated every year on (i) the main anabolic BVOC emission, that is, isoprene and methanol (ii) catabolic BVOC produced from isoprene and methanol and (iii) the ratio between catabolic BVOC and their precursors as an indicator of the oxidative pressure due to drought. These data allow to better understand the tolerance mechanisms of *Q. pubescens* to climate change.

## Materials and methods

### Experimental site

**Figure 1:**
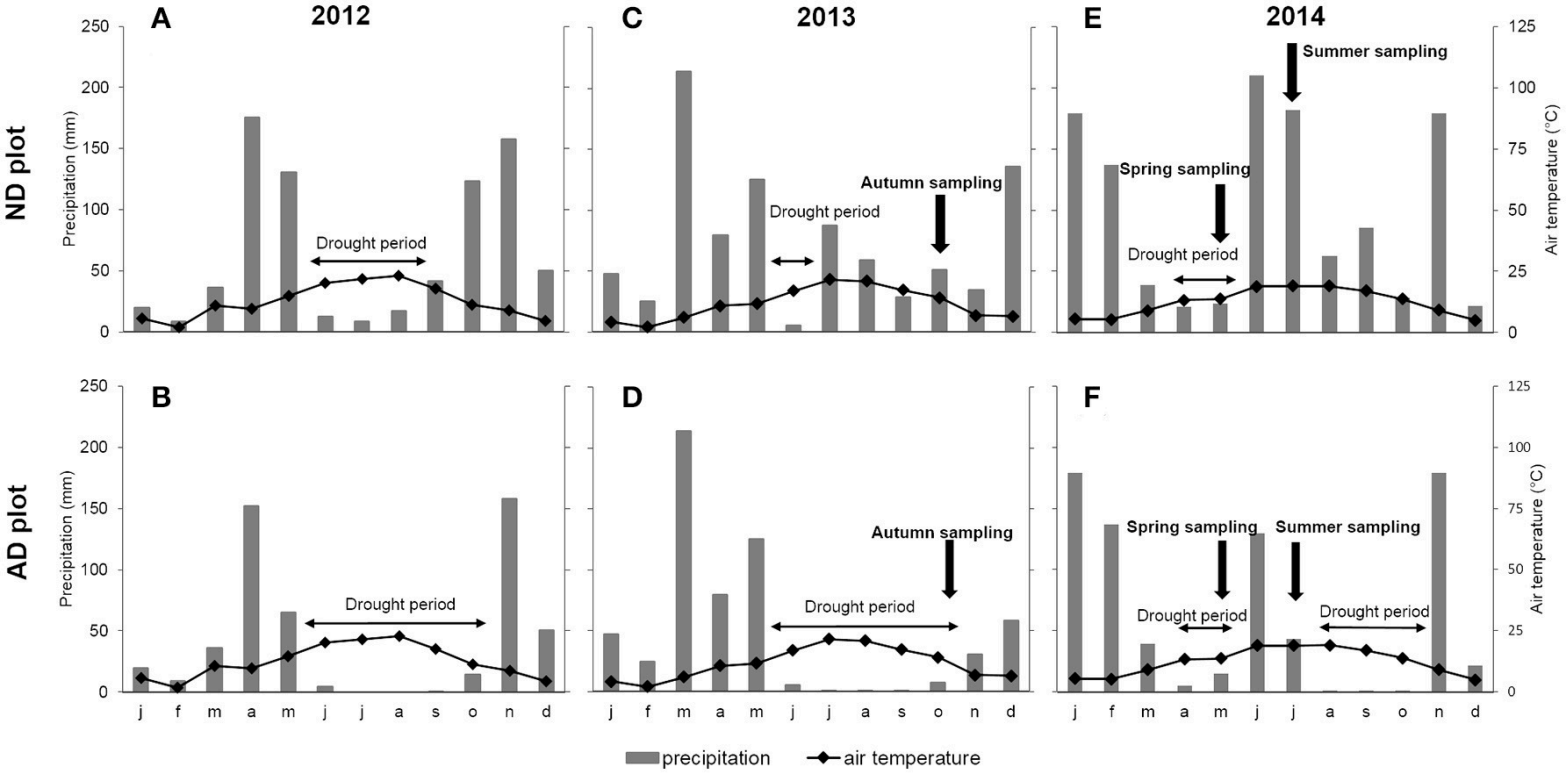
**Ombrothermic diagram of (A)** natural drought (ND) plot for 2012, **(B)** amplified drought (AD) plot for 2012, **(C)** ND plot for 2013, **(D)** AD plot in 2013, **(E)** ND plot for 2014 and **(F)** AD plot for 2014. Bars represent mean monthly precipitation (mm) and curves represent mean monthly temperature (°C).

This experiment was performed at the O_3_HP site (Oak Observatory at the Observatoire de Haute Provence, OHP), located in 60 km north from Marseille (5°42′44” E, 43°55′54” N), at an elevation of 650 m above mean sea level. The O_3_HP (955 m^2^), free from human disturbance for 70 years, consists of a homogeneous forest mainly composed of *Q. pubescens* (≈ 90% of the biomass and ≈ 75% of the trees). The remaining 10% of the biomass is mainly represented by *Acer monspessulanum* L. trees. The O_3_HP site was created in 2009 in order to study the impact of climate change on *Q. pubescens* forest ecosystem. Using a rainfall exclusion device (an automated monitored roof deployed during rain events) set up over part of the O_3_HP canopy, it was possible to reduce natural rain by 30% and to extend the drought period in an attempt to mimic the current climatic model projections for 2100 (Giorgi and Lionello, 2008; IPCC, 2013; Polade et al., 2014). Two plots were considered in the site; a plot receiving natural precipitations where trees grew under natural drought (ND, 300 m^2^) and a second plot submitted to amplified drought (AD, 232 m^2^). Rain exclusion on AD plot started on April 2012 and was continuously applied every year, principally, during the growth period. Ombrothermic diagrams indicated that the drought period was extended for 2 months in 2012, 4 months in 2013, and 3 months in 2014 for AD relative to ND (Figure 1). Data on cumulative precipitation (Figure S1 in Supplementary Files) showed that 35% of rain was excluded in 2012 (April 29th from to October 27th), 33% in 2013 (July 7th from to December 29th), 35.5% in 2014 (from April 8th to December 8th). This experimental set up involved a chronic drought for AD plot and in a lesser extent for ND plot (depending on the climatic conditions of the year). In this study, three measurement campaigns are presented from October 2013 to July 2014, that is, during part of the 2nd and 3rd year of AD, including an entire seasonal cycle: from October 14th to 28th 2013 for autumn, May 12th to 19th 2014 for spring and July 13th to 25th 2014 for summer. Five trees per plot were studied throughout the 3 seasons. These trees were already studied in 2012 and 2013 (Genard-Zielinski et al., 2015, personal communication from A.C. Genard-Zielinski). The sampling was performed at the branch-scale at the top of the canopy.

### Branch scale-sampling methods

Dynamic branch enclosures used for monitoring gas exchanges and BVOC were fully described in Genard-Zielinski et al. (2015) and only slight modifications described herein were made. Branches were enclosed in a ≈30 L PTFE (polytetrafluoroethylene) frame closed by a 50 μm thick PTFE film. Inlet air was introduced at 9 L.min^−1^ (insuring an air renewal time of only 3.33 min) using a PTFE pump (KNF N840.1.2FT.18®, Germany) and, ozone was removed from inlet air by placing PTFE filters impregnated with sodium thiosulfate (Na_2_S_2_O_3_) according to Pollmann et al. (2005). Since isoprene and methanol are oxidized by ozone under relatively high residence times only (1.3 days for isoprene and more than 4.5 years for methanol under controlled conditions) (Atkinson and Arey, 2003), the sampling conditions should be, by far, sufficient to impede BVOC oxidations due to ozone. The inlet air was dried using drierite before being analyzed by PTR-ToF-MS. A PTFE fan ensured a rapid mixing of the chamber air and a slight positive pressure within the enclosure enabled it to be held away from the leaves to avoid biomass damage. Environmental parameters (temperature, relative humidity, and photosynthetically active radiation or PAR) were continuously (every minute) monitored using a data logger (LI-COR 1400®; Lincoln, NE, USA) with a RHT probe placed inside the chamber (relative humidity and temperature, LI-COR 1400–04®, Lincoln, NE, USA) and a quantum sensor (PAR, LI-COR, PAR-SA 190®, Lincoln, NE, USA) placed outside the chamber. The latter sensor was set up and maintained horizontally. Branch enclosures were installed on the previous day before the first measurement and were continuously flushed with purified air. Air flow rates were controlled using mass flow controllers (MFC, Bronkhorst) and all tubing lines were PTFE-made.

### Ecophysiological parameters

Exchanges of CO_2_ and H_2_O from the enclosed branches were also continuously (every minute) measured using infrared gas analysers (IRGA 840A®, LI-COR). All gas exchanges were averaged between 12:00 and 15:00 (local time) where branches were the most physiologically active. Net photosynthesis (Pn, μmolCO_2_ .m^−2^.s^−1^) was calculated using equations described by von Caemmerer and Farquhar (1981) as follows:

$$\begin{array}{l} \text{Pn}=\frac{F{\;}^{*}\;\left(Cr-Cs\right)}{S}-CS{\;}^{*}\;E \end{array}$$

where F is the inlet air flow (mol.s^−1^), Cs and Cr are the sample and reference CO_2_ molar fraction respectively (ppm), S is the leaf surface (m^2^), Cs ∗ E is the fraction of CO_2_ diluted in water evapotranspiration and E (molH_2_O.m^−2^.s^−1^ then transformed in mmolH_2_O.m^−2^.s^−1^, afterward) is the transpiration rate calculated as follow:

$$\begin{array}{l} \text{E}=\frac{F{\;}^{*}\;\left(Ws-Wr\right)}{S{\;}^{*}\;\left(1-Ws\right)} \end{array}$$

where Ws and Wr are the sample and the reference H_2_O molar fraction respectively (molH_2_O.mol^−1^). Stomatal conductance (Gw, mmolH_2_O.m^−2^.s^−1^) was calculated using the following equation:

$$\begin{array}{l} \text{Gw}=\frac{E{\;}^{*}\;\left(1-\frac{\left(Ws+Wl\right)}{2}\right)}{Wl-Ws} \end{array}$$

where Wl is the molar concentration of water vapor within the leaf (molH_2_O.mol^−1^) calculated as follows:

$$\begin{array}{l} Wl=\frac{Vpsat}{P} \end{array}$$

where Vpsat is the saturated vapor pressure (kPa) and P is the atmospheric pressure (kPa). Water use efficiency (WUE, mmolCO_2_.molH_2_O^−1^) was calculated with the following equation:

$$\begin{array}{l} \text{WUE}=\frac{Pn}{E} \end{array}$$

Diurnal cycles for Pn and Gw were presented in Supplementary Files (Figure S2). Leaves from enclosed branches were directly collected after the sampling. Then, the surface of this leaves was assessed with a leaf area meter (AM350) to calculate physiological parameters on a leaf area basis. After that, leaves were lyophilized to assess the dry mass. Stem water potential was measured at midday (Ψm) for each season and at predawn (Ψpd) only for summer with a scholander pressure chamber (PMS instrument Co. Oregon USA).

### Quantification of BVOC

A commercial PTR-ToF-MS 8000 instrument (Ionicon Analytik GmbH, Innsbruck, Austria) was used for online measurements of BVOC (Tholl et al., 2006; Jordan et al., 2009). The reaction chamber pressure was fixed at 2.1 mbar, the drift tube voltage at 550 V and the drift tube temperature at 313 K corresponding to an E/N ratio (electric field strength over buffer gas number density) of ≈125 Td (1 Td = 10^−17^ V cm^2^). Each sample (AD—inlet air—ND—ambient air—catalyser) was sequentially monitored every hour during 15 min over 1 or 2 days using a multi-position common outlet flow path selector valve system (Vici) and a vacuum pump. Mass spectra were recorded up to m/z 500 at 1 min integration time. BVOC targeted in this study and their corresponding ions include formaldehyde (m/z 31.018), methanol (m/z 33.033), isoprene (m/z 41.038, 69.069), and MACR+MVK+ISOPOOH (m/z 71.049, these three compounds were detected with the same ion). BVOC mixing ratios are calculated using the proton transfer rate constants k (cm^3^.s^−1^) reported by Cappellin et al. (2012), the reaction time in the drift tube and the experimentally determined ion transmission efficiency. The relative ion transmission efficiency was assessed using a standard gas calibration mixture (TO-14A Aromatic Mix, Restek Corporation, Bellefonte, USA) (100 ± 10 ppb in Nitrogen). Formaldehyde sensitivity dependence to air water content, was taken into account and corrected according to Vlasenko et al. (2010) method.

BVOC emissions rates (ER) at noon (between 12:00 and 15:00 local time), where emissions were the strongest, were calculated by considering the BVOC concentrations at the inlet and outlet of the PTFE chamber such as:

$$\begin{array}{l} ER=\frac{\left(Q_{0}{\;}^{*}\;\left(C_{out}-C_{in}\right)\right)}{B} \end{array}$$

where ER is expressed in μgC.${\text{g}}_{\text{DM}}^{-1}$.h^−1^, Q_0_ is the flow rate of the air introduced into the chamber (L.h^−1^), C_out_ and C_in_ are the concentrations in the outflowing and inflowing air respectively (μgC.L^−1^) and B is the total dry biomass matter (g_DM_). The ER expressed in μgC.m^−2^.h^−1^ were also calculated and presented in Supplementary Files (Figure S3). Moreover, diurnal cycle of BVOCs were presented in Supplementary Files (Figures S4, S5). The ratio of the catabolic BVOC emission rates (MACR+MVK+ISOPOOH, formaldehyde) to their respective anabolic precursor emission rates (isoprene, methanol) was calculated as follows:

$$\begin{array}{l} \text{E}{\text{R}}_{\text{catabolic}}/\text{E}{\text{R}}_{\text{anabolic}}\;=\frac{ER_{catabolic}\;*\;100}{ER_{anabolic}} \end{array}$$

This parameter is an indirect indicator of oxidative pressure, that is, increasing ERcatabolic/ERanabolic ratio would reflects an enhanced oxidation of anabolic BVOC within leaves through catabolic processes. Moreover, in order to understand isoprene emission changes, the allocation of the assimilated CO_2_ in isoprene emissions was calculated according to the following equation:

$$\begin{array}{l} CO_{2}\;allocation\;in\;isoprene\;emissions=\frac{E{R_{isoprene}}^{*}100}{Pn}\left(4\right) \end{array}$$

where ER isoprene used in this equation was expressed in μgC.m^−2^.h^−1^ and Pn was transformed also in μgC.m^−2^.h^−1^.

### Data treatment

Statistical analyses were performed with STATGRAPHICS® centurion XV (Statpoint Technologies, Inc.). To evaluate the effect of AD and season on the ecophysiological parameters and BVOC emissions, two-way repeated measure ANOVA followed by Tukey *post-hoc* tests were performed, after having checked the normality and homoscedasticity of the data set. When interactions between seasonality and drought occurred, one-way repeated ANOVA followed by Tukey *post-hoc* tests were performed to evaluate the effect of season on AD and ND separately, and Student tests to evaluate differences between ND and AD during each season. Ψpd, only available for summer, was analyzed using Student test whereas Ψm was analyzed in the same way than BVOC emissions. Moreover, Pearson's correlations were made between the anabolic ER and catabolic ER.

## Results

### Ecophysiological parameters

**Figure 2:**
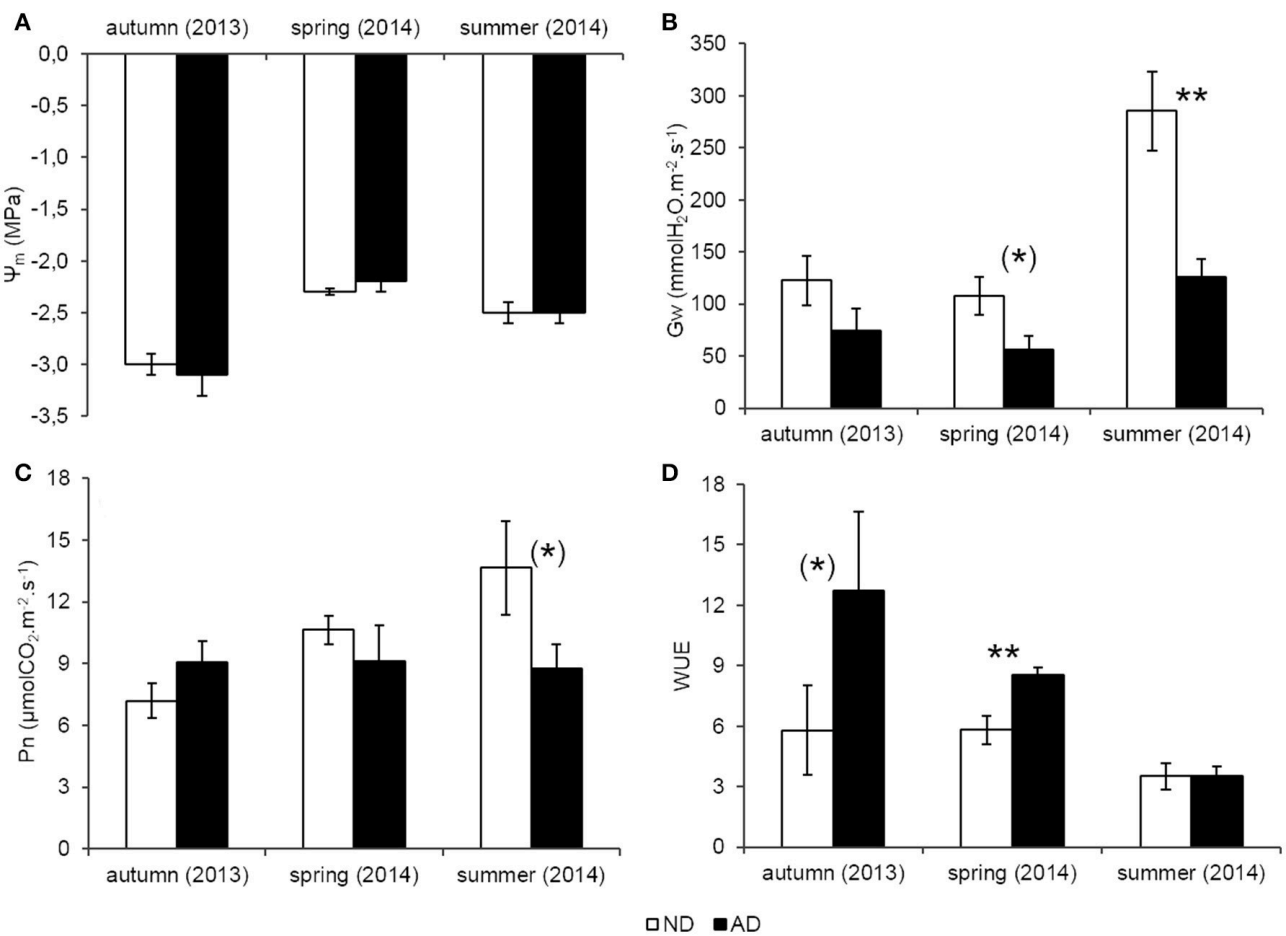
**(A)** Leaf water potential at midday (Ψm, MPa) **(B)** Stomatal conductance (Gw, mmolH_2_O.m^−2^.s^−1^) **(C)** Net photosynthesis (Pn, μmolCO_2_.m^−2^.s^−1^) **(D)** Water use efficiency (WUE) according to natural (ND) and amplified drought (AD) over the seasonal cycle. Asterisks denote significant differences between treatments for each season with Student tests with (^*^) = 0.05 < *P* < 0.1 and ^**^ = 0.001 < *P* < 0.01. Values are means ± S.E and *n* = 5.

The water status of trees at midday, indicated by Ψm, was not decreased with amplified drought (AD, Figure 2A) and dropped below -2 MPa for both treatments whatever the season. By contrast, the predawn water potential (Ψpd) was significantly lower under AD (Student test, *P* < 0.05, −0.61 ± 0.02 MPa for ND and −0.85 ± 0.10 MPa for AD, data not shown), indicating that the water availability in the AD plot decreased at predawn but this difference was blurred during the day. Gw decreased with AD, especially in summer (−56%, *P* < 0.001) and to a lesser extent in spring (−47%, 0.05 < *P* < 0.1, Figure 2B). Thus, *Q. pubescens*, with AD, closed its stomata leading to a decrease of Pn by 36% in summer but it is only a trend (Figure 2C, 0.05 < *P* < 0.1), while it did not reduce Pn in spring. Trees from the AD plot used water more efficiently for CO_2_ assimilation in spring (WUE was 34% higher in the AD plot than the ND plot, Figure 2D, 0.05 < *P* < 0.1). By contrast, in summer, water was used in the same way by trees growing in both plots.

### Anabolic BVOC

**Figure 3:**
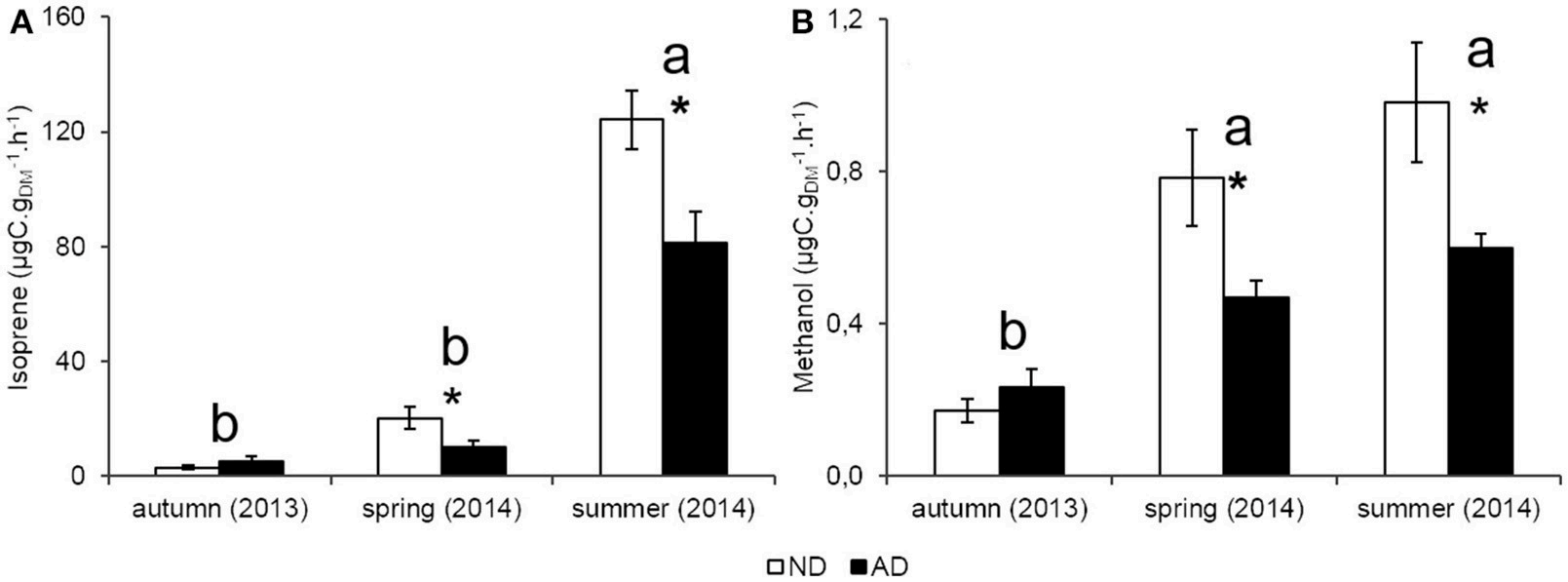
**Anabolic emissions rate (ER, μgC.${\text{g}}_{\text{DM}}^{-1}$.h^**−1**^) of (A)** isoprene and **(B)** methanol according to natural (ND) and amplified drought (AD) over the seasonal cycle. Asterisks denote significant differences between treatments according to a two-way repeated measures ANOVA with ^*^ = 0.01 < *P* < 0.05 and letters denote significant differences among seasons with Tukey *post-hoc* test with a>b. Values are means ± S.E and *n* = 5.

**Figure 4:**
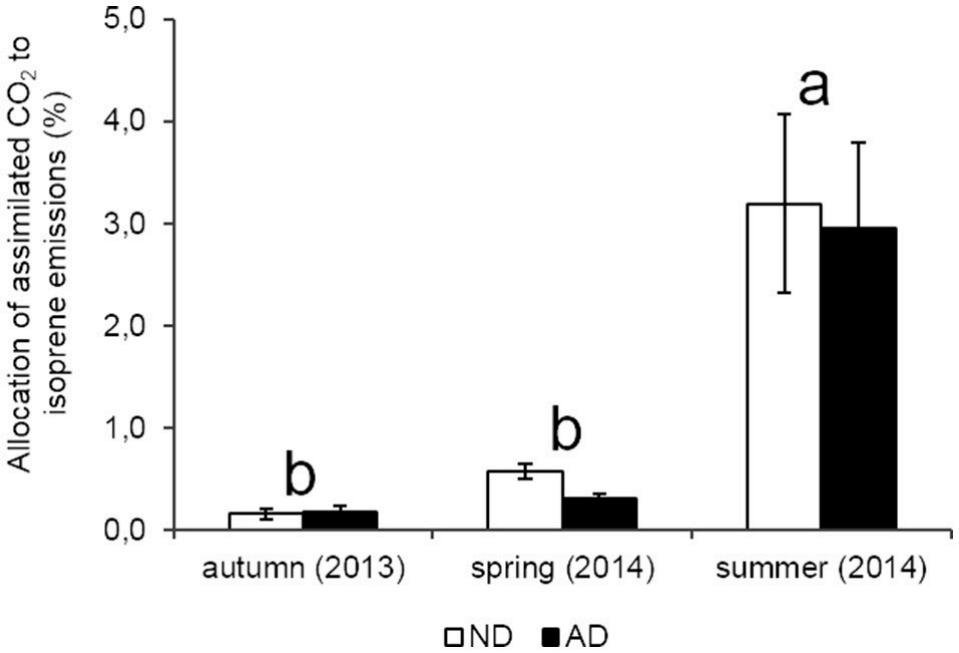
**Allocation of assimilated CO_**2**_ to isoprene emissions (%) according to natural (ND) and amplified drought (AD) over the seasonal cycle**. Letters denoted significant differences between seasons according to a two-way repeated measures ANOVA with Tukey *post-hoc* test with a>b. Values are means ± S.E and *n* = 5.

Seasonality had a strong effect on isoprene emission rates (*P* < 0.001) in both plots with a peak of emissions in summer (124.3 and 81.1 μgC.${\text{g}}_{\text{DM}}^{-1}$.h^−1^ for ND and AD plots, respectively), when PAR and temperatures were the highest. Isoprene emission rates in spring ranged between 20.3 and 10.2 μgC.${\text{g}}_{\text{DM}}^{-1}$.h^−1^ under ND and AD respectively, and were not significantly different from those of autumn (2.9 and 5.2 μgC.${\text{g}}_{\text{DM}}^{-1}$.h^−1^ for ND and AD plots, respectively). Yet, isoprene emission rates were impacted by AD (Figure 3A, *P* < 0.01) as they decreased by 35% in summer and by 50% in spring compared to ND plot. However, there was no significant effect of AD in autumn. Regarding the allocation of assimilated CO_2_ to isoprene emissions, AD had no effect (Figure 4, *P* > 0.05). Nevertheless, such allocation was clearly favored in summer in both plots (*P* < 0.001). Indeed, ~3% of the assimilated CO_2_ was released as isoprene in summer whereas in the other seasons, this value was less than 1%.

Methanol emission rates (Figure 3B) were highly sensitive to seasonality (*P* < 0.001) with the lowest emissions occurring in autumn (0.17 and 0.23 μgC.${\text{g}}_{\text{DM}}^{-1}$.h^−1^ for ND and AD respectively) compared to the other seasons when methanol emissions were higher or close to 0.5 μgC.${\text{g}}_{\text{DM}}^{-1}$.h^−1^ for both merged plots. AD also had a significant effect on methanol emissions rates (*P* < 0.01), especially in spring and summer, where emissions decreased by 40%. As for isoprene, AD had no effect on methanol emissions rates in autumn.

### Catabolic BVOC

**Figure 5:**
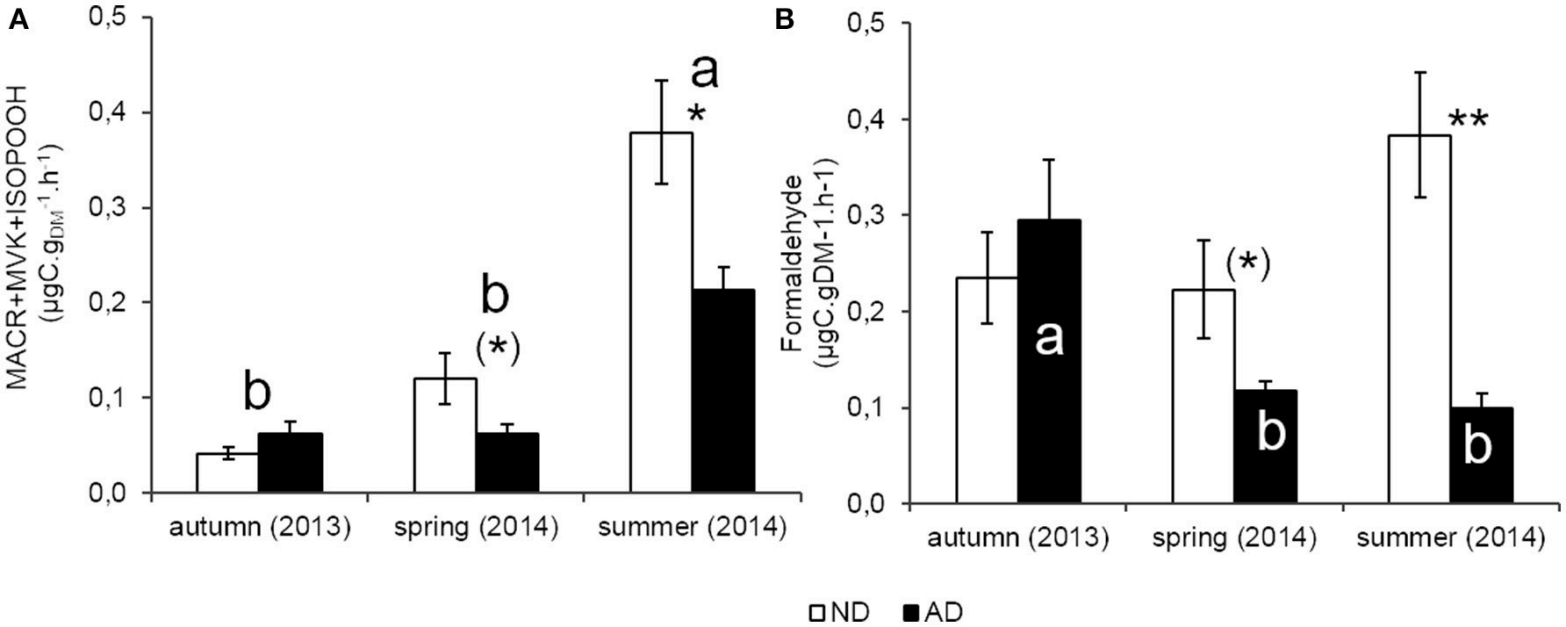
**Catabolic BVOC emissions rate (μgC.${\text{g}}_{\text{DM}}^{-1}$.h^**−1**^) of (A)** isoprene catabolic products (MACR/MVK/ISOPOOH) and **(B)** methanol catabolic product (Formaldehyde) according to natural (ND) and amplified drought (AD) over the seasonal cycle. Asterisks denote significant differences between treatments according to a two-way repeated measures ANOVA with (^*^) = 0.05 < *P* < 0.1; ^*^ = 0.01 < *P* < 0.05; ^**^ = 0.001 < *P* < 0.01 and letters denote significant differences among seasons according to a Tukey *post-hoc* test with a>b. For formaldehyde, there is only a season effect on AD plot. Values are means ± S.E and *n* = 5.

MACR+MVK+ISOPOOH emissions rates (catabolic products of isoprene) showed, as isoprene, a clear seasonal course (Figure 5A, *P* < 0.001) with the highest emissions in summer (0.38 and 0.21 μgC.${\text{g}}_{\text{DM}}^{-1}$.h^−1^, for ND and AD plots respectively) compared to the other seasons where emission rates were lower or close to 0.1 μgC.${\text{g}}_{\text{DM}}^{-1}$.h^−1^ for both merged plots. Moreover, as isoprene, seasonality remained the same with AD. By contrast, MACR+MVK+ISOPOOH emission rates were impacted by AD (*P* < 0.01) which decreased emissions by 44% in summer (*P* < 0.05) and, by 48% in spring (0.1 > *P* > 0.05). Nevertheless, AD did not modify catabolic products of isoprene emissions as in autumn.

The effect of seasonality on formaldehyde emissions was less clear than for the other compounds (Figure 5B). Results of two-way repeated measure ANOVA did not show a seasonality effect (*P* > 0.05) but there was a significant interaction between both factors. The strongest emissions were observed in autumn (0.29 μgC.${\text{g}}_{\text{DM}}^{-1}$.h^−1^) compared to the other seasons, when emissions were lower to close to 0.1 μgC.${\text{g}}_{\text{DM}}^{-1}$.h^−1^. Thus, AD modified the seasonality pattern of formaldehyde emission rates unlike the other compounds. Furthermore, these emissions were impacted by AD especially in summer (*P* < 0.01) with a decrease of 74%, and to a lesser extent in spring with 48% of reduction (0.1 > *P* > 0.05).

### Relationship between catabolic and anabolic BVOC

**Figure 6:**
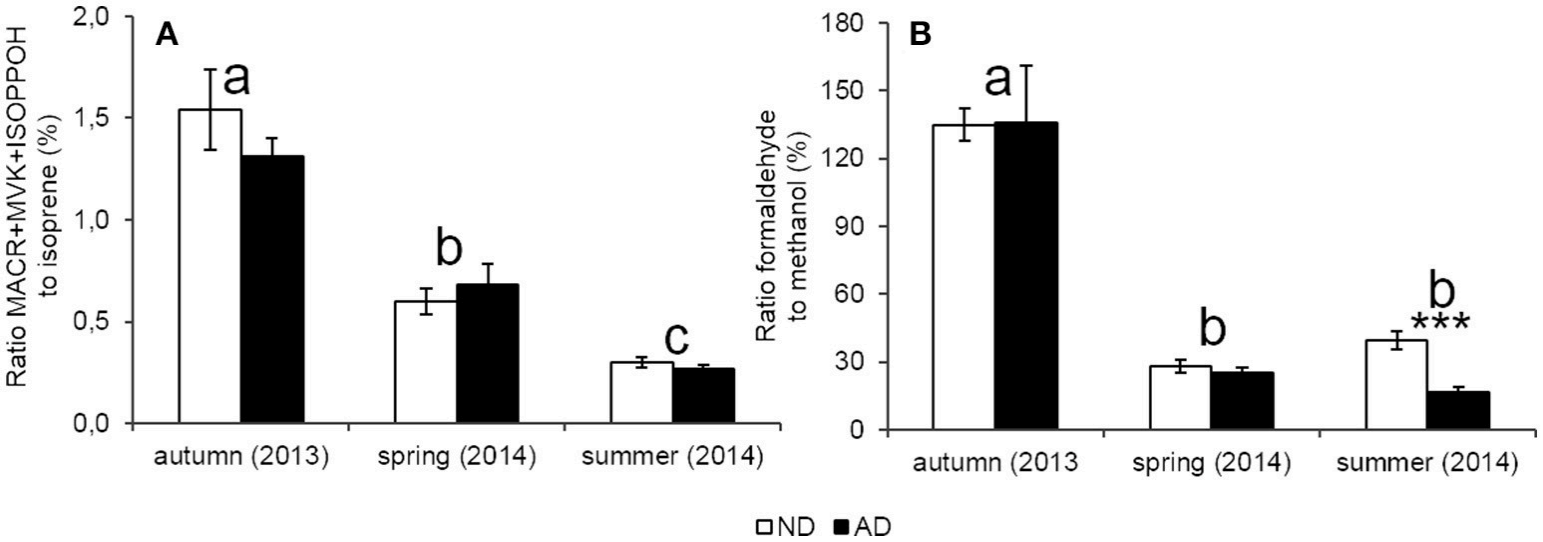
**Ratio between catabolic BVOC and their precursors (%) (A)** catabolic way of isoprene **(B)** catabolic way of methanol according to natural (ND) and amplified drought (AD) over the seasonal cycle. Asterisks denote significant differences between treatments according to a two-way repeated measures ANOVA with ^***^ = *P* < 0.001 and letters denote significant differences among seasons according to a Tukey *post-hoc* test with a>b>c. Values are means ± S.E and *n* = 5.

The ERMACR+MVK+ISOPOOH/ERisoprene ratio, which can reflect the degree of oxidation within leaves, showed a strong seasonality (Figure 6A, *P* < 0.001) with the highest ratio in autumn, intermediate ratio in spring and the lowest in summer. However, these ratios were similar under AD and ND (*P* > 0.05). Likewise, the ERformaldehyde/ERmethanol ratio was not impacted by AD (Figure 6B, *P* > 0.05). Moreover, a strong effect of seasonality was observed (*P* < 0.001) with larger oxidation rates (i.e., higher ratios) in autumn with regard to other seasons. Moreover, formaldehyde emission rates were higher than methanol emissions rates, and thereby, the ERformaldehyde/ERmethanol ratio, in autumn, was superior to 100%.

Isoprene and MACR+MVK+ISOOOH emission rates were well-correlated at any season and whatever the drought treatment (Table 1). Isoprene emission rates explained more than 85% of MACR+MVK+ISOPOOH emission rates whatever the season or the drought treatment (*R*^2^ > 0.85). By contrast, more reserved results were obtained with correlation between methanol and formaldehyde, especially in autumn when methanol emission rates explained less than 25 and 41% for ND and AD respectively, of formaldehyde emission rates. For the other seasons, correlations were stronger than but not as strong as between isoprene and MACR+MVK+ISOPOOH emission rates.

**Table 1:** **R-squared (***R***^**2**^) coefficients and, in parentheses, the slope of linear correlations between anabolic and catabolic BVOC emissions rate according to natural (ND) and amplified drought (AD) over the seasonal cycle**.

	**Isoprene-MACR**+**MVK+ ISOPOOH**		**Methanol-Formaldehyde**	
	**ND**	**AD**	**ND**	**AD**
Autumn (2013)	0.94 (0.01)	0.95 (0.01)	0.25 (0.66)	0.41 (1.03)
Spring (2014)	0.91 (0.01)	0.96 (0.01)	0.73 (0.24)	0.70 (0.18)
Summer (2014)	0.86 (0.002)	0.88 (0.002)	0.74 (0.33)	0.54 (0.28)

*All the correlations are significant with P < 0.001*.

## Discussion

### Ecophysiological parameters

Results showed that Ψm remained unchanged with AD. By contrast, Ψpd supported the hypothesis that the restricted precipitations in the AD reduced water availability compared to ND. This species has a high hydraulic stem efficiency (Nardini and Pitt, 1999) which is likely to allow for compensation of water loss by an equal amount of water uptake during the day, enabling *Q. pubescens* to maintain its midday water status even under AD. The poor water availability under AD induced stomata closure both in spring and summer. Despite stomata closure, Pn was only slightly reduced in summer, while in spring, the higher WUE allowed to maintain Pn. Increasing the efficiency of water used to fix CO_2_ allows *Q. pubescens* to reduce the effect of AD. This results indicated that *Q. pubescens* can cope with a 30% reduction of natural rain involving a moderate stress in this species compared with trees under ND (Niinemets, 2010a). It is likely that *Q. pubescens* developed such a strategy to be competitive under prolonged water stress periods. Other authors have showed under controlled experimental conditions that water scarcity induced a strong decay of Pn in young *Q. pubescens* saplings and, at the same time, Gw went down (Brüggemann and Schnitzler, 2002; Gallé et al., 2007; Genard-Zielinski et al., 2014). However, in these studies, the water status, defined by Ψpd, was very low compared with our experiment (below –3 MPa against –0.85 MPa). *Q. pubescens* Ψpd can actually drop below –4 MPa before the first signs of real decay (Damesin and Rambal, 1995), which confirms the implementation of sustainable stress for *Q. pubescens* during our experiment even after 3 consecutive years of AD.

### Anabolic BVOC

Several mechanistic studies have supported the role of isoprene as a protective metabolite against the oxidative damage induced by environmental stresses (Vickers et al., 2009; Velikova et al., 2011; Pollastri et al., 2014). In agreement, numerous studies have reported an isoprene emission sensitivity to moderate water or heat stress (Behnke et al., 2007; Fortunati et al., 2008; Genard-Zielinski et al., 2014). It is also already known that terpenes (mainly isoprene and monoterpenes) follow a seasonal trend with the highest emissions in summer (Goldstein et al., 1998; Pio et al., 2005). In our study, a clear seasonality appeared with the largest isoprene emissions in summer when the oxidative stress was strongest due to climatic conditions. This observation confirms the possible role of isoprene in the protection of photosynthetic apparatus against oxidative damage in *Q. pubescens*.

In the first year of AD, it was observed an increase of isoprene emissions in our experiment (personal communication from A.C. Génard-Zielinski), especially in summer (August 2012) with a simultaneous decrease of Gw and Pn. By contrast, after 3 years of drought, isoprene emissions were reduced by 50 and 35% (in spring and summer respectively) whereas we expected an increase of these emissions according to the results obtained in 2012. This decrease cannot be explained by stomata closure alone as isoprene can also be emitted through the cuticle (Monson and Fall, 1989; Sharkey and Yeh, 2001). The most reliable explanation is that the reduction of Pn observed in our study involving a lower carbon availability in trees. This is confirmed by the stability of the carbon allocation in isoprene with 2.95 and 3.19% of the carbon assimilated through Pn invested in isoprene production, in summer for AD and ND, respectively. Moreover, this poor allocation of the assimilated carbon indicated that trees did not suffer a strong water stress. Indeed, under optimal conditions, this allocation usually ranges between 2 and 5% and can reach between 10 and 50% under strong stressed conditions (Brilli et al., 2007; Genard-Zielinski et al., 2014). These results suggest that *Q. pubescens* was able to sustain a 30% reduction of natural rain, after three consecutive years of application, without increasing the investment in isoprene emissions; contrary to what had been observed during the first year of this experiment (personal communication from A.C. Génard-Zielinski). It is possible that isoprene is only a response to short-term water stress and when the drought becomes chronic, other antioxidant metabolites are favored but this hypothesis deserves ongoing research.

Methanol emission rates followed a seasonal course with high emissions in spring and summer compared to autumn as previously observed for other species (Schade and Goldstein, 2006; Lappalainen et al., 2009; Hu et al., 2011). Methanol was especially released during the growth period (elongation and maturation of leaves) when demethylation of pectin in the primary cell walls occurred (Hüve et al., 2007; Oikawa and Lerdau, 2013). AD did not modify the seasonality but had a slight effect on methanol emissions with an emission decrease in spring and summer that could probably be due to the stomatal closure observed in these seasons. Indeed, since oxygenated BVOC have a higher water affinity, they are mainly released during water exchanges and hence stomatal aperture (Niinemets et al., 2004).

### Catabolic BVOC and their relationship with their respective anabolic BVOC precursors

MACR+MVK+ISOPOOH emission rates followed the same pattern of seasonality than isoprene emission rates with the strongest emissions observed in summer. The effect of AD is also the same than isoprene emissions with a trend to decrease under AD in spring and summer. These results and the high correlations between MACR+MVK+ISOPOOH and isoprene emissions, suggest that MACR+MVK+ISOPOOH formation in plants originated only from isoprene oxidations within leaf tissues. However, there are still large uncertainties about the MACR+MVK+ISOPOOH origin within leaves (Guenther, 2013) and this hypothesis needs to be further investigated. Seasonality had a strong effect on ERMACR+MVK+ISOPOOH/ERisoprene ratio with the highest values occurring in autumn. Although, at this time of the year, leaf senescence promotes lipid peroxidations which is a known source of ROS (Bhattacharjee, 2005). These ratios did not show any significant difference between ND and AD probably due to the slight intensity of AD as previously discussed. The ratio of isoprene oxidized in MACR+MVK+ISOPOOH can vary strongly across species. Jardine et al. (2012) found a ratio of 25% between isoprene and MACR+MVK+ISOPOOH in controlled conditions for *Phytolacca dioica* L. whereas we only found 1.54% for ND and 1.30% for AD in autumn. These differences could be explained by leaf temperature during measurement with 47°C for Jardine et al. (2012) and only 30°C during the warmest season of our study.

Formaldehyde and methanol emission rates did not follow the same seasonality unlike isoprene and MACR+MVK+ISOPOOH emission rates. Indeed, higher formaldehyde emissions were observed in autumn only in the AD plot whereas higher methanol emissions were observed during the growth period. Nevertheless, formaldehyde and methanol emissions rates were impacted by AD in the same way; especially in spring and summer, when a strong decrease of emissions occurred. As formaldehyde is also an oxygenated BVOC, we can assume that, as for methanol, stomatal aperture is a driver of its emission. Moreover, in autumn, methanol and formaldehyde showed a relatively weak correlation and formaldehyde emissions represent more than 100% methanol emissions. These results suggest that methanol and formaldehyde emissions were not strongly bounded in autumn with other sources of formaldehyde formation occurring in *Q. pubescens* during this season such as the dissociation of 5,10-methylene-tetrahydrofolate, oxidative demethylation reactions or the glyoxylate decarboxylation or isoprene oxidations (Atkinson and Arey, 2003; Seco et al., 2007; Oikawa and Lerdau, 2013). Moreover, Holzinger et al. (2000) who worked on *Q. ilex*, found that ratio of formaldehyde to methanol was only equal to 4%, whereas we found, for all seasons, a ratio always higher. This difference can be due to the implementation of water stress. Indeed, Holzinger et al. (2000) worked on flooding stress in roots whereas we worked on water stress.

## Conclusion

Anabolic and catabolic BVOC emissions were observed to be strongly seasonal dependent. Moreover, all anabolic and catabolic emission rates decreased, especially in summer. While the decrease of oxygenated BVOC (methanol, formaldehyde, and isoprene catabolic products) can, probably, be attributed to stomata closure, isoprene emission decrease is rather related to the decrease of Pn. Catabolic emissions and the ratios could be an indicator of oxidative pressure but it depends on the relationship between catabolic BVOC and their precursors. Our results highlighted that MACR+MVK+ISOPOOH could be a potential good indicator unlike formaldehyde. Other catabolic BVOC could be used as indicators of oxidative pressure such as acetaldehyde (catabolic products of ethanol) or the green leaf volatile compounds (GLVs, catabolic products of cell membranes) but that requires some further research.

## Author contributions

EO and CF designed the research; AS, EO, and CF conducted the research; AS, EO, HW, BT, CL, and CF collected and analyzed the data; AS, EO, HW, BT, CB, and CF wrote the manuscript.

### Conflict of interest statement

The authors declare that the research was conducted in the absence of any commercial or financial relationships that could be construed as a potential conflict of interest.

## References

[B1] Arneth A. Monson R. Schurgers G. Niinemets Ü. Palmer P. (2008). Why are estimates of global terrestrial isoprene emissions so similar (and why is this not so for monoterpenes)? Atmos. Chem. Phys. 8, 4605–4620. 10.5194/acp-8-4605-2008

[B2] Atkinson R. Arey J. (2003). Gas-phase tropospheric chemistry of biogenic volatile organic compounds: a review. Atmos. Environ. 37(Suppl. 2), 197–219. 10.1016/s1352-2310(03)00391-1

[B3] Behnke K. Ehlting B. Teuber M. Bauerfeind M. Louis S. Hänsch R.. (2007). Transgenic, non-isoprene emitting poplars don't like it hot. Plant J. 51, 485–499. 10.1111/j.1365-313X.2007.03157.x 17587235

[B4] Bhattacharjee S. (2005). Reactive oxygen species and oxidative burst: roles in stress, senescence and signal. Curr. Sci. India 89, 1113–1121.

[B5] Brilli F. Barta C. Fortunati A. Lerdau M. Loreto F. Centritto M. (2007). Response of isoprene emission and carbon metabolism to drought in white poplar (*Populus alba*) saplings. New Phytol. 175, 244–254. 10.1111/j.1469-8137.2007.02094.x 17587373

[B6] Brüggemann N. Schnitzler J. P. (2002). Comparison of isoprene emission, intercellular isoprene concentration and photosynthetic performance in water-limited oak (*Quercus pubescens* Willd. and *Quercus robur* L.) Saplings. Plant Biol. 4, 456–463. 10.1055/s-2002-34128

[B7] Brzostek E. R. Dragoni D. Schmid H. P. Rahman A. F. Sims D. Wayson C. A.. (2014). Chronic water stress reduces tree growth and the carbon sink of deciduous hardwood forests. Glob. Change Biol. 20, 2531–2539. 10.1111/gcb.12528 24421179

[B8] Calogirou A. Larsen B. Kotzias D. (1999). Gas-phase terpene oxidation products: a review. Atmos. Environ. 33, 1423–1439. 10.1016/S1352-2310(98)00277-5

[B9] Cappellin L. Karl T. Probst M. Ismailova O. Winkler P. M. Soukoulis C.. (2012). On quantitative determination of volatile organic compound concentrations using proton transfer reaction time-of-flight mass spectrometry. Environ. Sci. Technol. 46, 2283–2290. 10.1021/es203985t 22296026

[B10] Damesin C. Rambal S. (1995). Field study of leaf photosynthetic performance by a Mediterranean deciduous oak tree (*Quercus pubescens*) during a severe summer drought. New Phytol. 131, 159–167. 10.1111/j.1469-8137.1995.tb05717.x

[B11] Fall R. (2003). Abundant oxygenates in the atmosphere: a biochemical perspective. Chem. Rev. 103, 4941–4952. 10.1021/cr0206521 14664637

[B12] Folkers A. Hüve K. Ammann C. Dindorf T. Kesselmeier J. Kleist E.. (2008). Methanol emissions from deciduous tree species: dependence on temperature and light intensity. Plant Biol. 10, 65–75. 10.1111/j.1438-8677.2007.00012.x 18211548

[B13] Fortunati A. Barta C. Brilli F. Centritto M. Zimmer I. Schnitzler J. P.. (2008). Isoprene emission is not temperature-dependent during and after severe drought-stress: a physiological and biochemical analysis. Plant J. 55, 687–697. 10.1111/j.1365-313X.2008.03538.x 18445130

[B14] Funk J. Mak J. Lerdau M. (2004). Stress-induced changes in carbon sources for isoprene production in *Populus deltoides*. Plant Cell Environ. 27, 747–755. 10.1111/j.1365-3040.2004.01177.x

[B15] Gallé A. Haldimann P. Feller U. (2007). Photosynthetic performance and water relations in young pubescent oak (*Quercus pubescens*) trees during drought stress and recovery. New Phytol. 174, 799–810. 10.1111/j.1469-8137.2007.02047.x 17504463

[B16] Genard-Zielinski A.-C. Boissard C. Fernandez C. Kalogridis C. Lathière J. Gros V. (2015). Variability of BVOC emissions from a Mediterranean mixed forest in southern France with a focus on *Quercus pubescens*. Atmos. Chem. Phys. Discuss. 14, 17225–17261. 10.5194/acp-15-431-2015

[B17] Genard-Zielinski A.-C. Orme-o E. Boissard C. Fernandez C. (2014). Isoprene emissions from downy oak under water limitation during an entire growing season: what cost for growth? PLoS ONE 9:e112418. 10.1371/journal.pone.0112418 25383554 PMC4226567

[B18] Giorgi F. Lionello P. (2008). Climate change projections for the Mediterranean region. Glob. Planet. Change 63, 90–104. 10.1016/j.gloplacha.2007.09.005

[B19] Goldstein A. H. Goulden M. L. Munger J. W. Wofsy S. C. Geron C. D. (1998). Seasonal course of isoprene emissions from a midlatitude deciduous forest. J. Geophys. Res. 103, 31045–31056. 10.1029/98JD02708

[B20] Guenther A. (2013). Biological and chemical diversity of biogenic volatile organic emissions into the atmosphere. Int. Sch. Res. Not. 2013:786290 10.1155/2013/786290

[B21] Guenther A. Hewitt C. N. Erickson D. Fall R. Geron C. Graedel T. (1995). A global model of natural volatile organic compound emissions. J. Geophys. Res. 100, 8873–8892. 10.1029/94JD02950

[B22] Harrison S. P. Morfopoulos C. Dani K. Prentice I. C. Arneth A. Atwell B. J.. (2013). Volatile isoprenoid emissions from plastid to planet. New Phytol. 197, 49–57. 10.1111/nph.12021 23145556

[B23] Holzinger R. Sandoval-Soto L. Rottenberger S. Crutzen P. Kesselmeier J. (2000). Emissions of volatile organic compounds from *Quercus ilex* L. measured by proton transfer reaction mass spectrometry under different environmental conditions. J. Geophys. Res. 105, 20573–20579. 10.1029/2000JD900296

[B24] Hu L. Millet D. Mohr M. Wells K. Griffis T. Helmig D. (2011). Sources and seasonality of atmospheric methanol based on tall tower measurements in the US Upper Midwest. Atmos. Chem. Phys. 11, 11145–11156. 10.5194/acp-11-11145-2011

[B25] Hüve K. Christ M. Kleist E. Uerlings R. Niinemets Ü. Walter A.. (2007). Simultaneous growth and emission measurements demonstrate an interactive control of methanol release by leaf expansion and stomata. J. Exp. Bot. 58, 1783–1793. 10.1093/jxb/erm038 17374874

[B26] IPCC (2013). Contribution of Working Group I to the Fifth Assessment Report of the Intergovernmental Panel on Climate Change. Cambridge: Cambridge Univeristy Press.

[B27] Jacob D. J. Field B. D. Li Q. Blake D. R. de Gouw J. Warneke C. (2005). Global budget of methanol: constraints from atmospheric observations. J. Geophys. Res. 110:D08303 10.1029/2004JD005172

[B28] Jardine K. J. Meyers K. Abrell L. Alves E. G. Serrano A. M. Y. Kesselmeier J.. (2013). Emissions of putative isoprene oxidation products from mango branches under abiotic stress. J. Exp. Bot. 64, 3669–3679. 10.1093/jxb/ert202 23881400 PMC3745727

[B29] Jardine K. J. Monson R. K. Abrell L. Saleska S. R. Arneth A. Jardine A. (2012). Within-plant isoprene oxidation confirmed by direct emissions of oxidation products methyl vinyl ketone and methacrolein. Glob. Change Biol. 18, 973–984. 10.1111/j.1365-2486.2011.02610.x

[B30] Jordan A. Haidacher S. Hanel G. Hartungen E. Märk L. Seehauser H. (2009). A high resolution and high sensitivity proton-transfer-reaction time-of-flight mass spectrometer (PTR-TOF-MS). Int. J. Mass Spectrom. 286, 122–128. 10.1016/j.ijms.2009.07.005

[B31] Kalogridis C. Gros V. Sarda-Esteve R. Langford B. Loubet B. Bonsang B. (2014). Concentrations and fluxes of isoprene and oxygenated VOCs at a French Mediterranean oak forest. Atmos. Chem. Phys. 14, 10085–10102. 10.5194/acp-14-10085-2014

[B32] Karl T. Guenther A. Spirig C. Hansel A. Fall R. (2003). Seasonal variation of biogenic VOC emissions above a mixed hardwood forest in northern Michigan. Geophys. Res. Lett. 30, 2186 10.1029/2003GL018432

[B33] Keenan T. Niinemets Ü. Sabate S. Gracia C. Pe-uelas J. (2009). Process based inventory of isoprenoid emissions from European forests: model comparisons, current knowledge and uncertainties. Atmos. Chem. Phys. Discuss. 9, 6147–6206. 10.5194/acpd-9-6147-2009

[B34] Kotzias D. Konidari C. Sparta C. (1997). Volatile Carbonyl Compounds of Biogenic ORIGIN. Emission and concentration in the Atmosphere. Biogenic Volatile Organic Carbon Compounds in the Atmosphere. Amsterdam SPB Academic Publishing.

[B35] Kreuzwieser J. Schnitzler J. P. Steinbrecher R. (1999). Biosynthesis of organic compounds emitted by plants. Plant Biol. 1, 149–159. 10.1111/j.1438-8677.1999.tb00238.x

[B36] Lappalainen H. Sevanto S. Bäck J. Ruuskanen T. Kolari P. Taipale R. (2009). Day-time concentrations of biogenic volatile organic compounds in a boreal forest canopy and their relation to environmental and biological factors. Atmos. Chem. Phys. 9, 5447–5459. 10.5194/acp-9-5447-2009

[B37] Lavoir A. V. Staudt M. Schnitzler J. P. Landais D. Massol F. Rocheteau A. (2009). Drought reduced monoterpene emissions from the evergreen Mediterranean oak *Quercus ilex*: results from a throughfall displacement experiment. Biogeosciences 6, 1167–1180. 10.5194/bg-6-1167-2009

[B38] Lichtenthaler H. K. (1999). The 1-deoxy-D-xylulose-5-phosphate pathway of isoprenoid biosynthesis in plants. Annu. Rev. Plant Biol. 50, 47–65. 10.1146/annurev.arplant.50.1.47 15012203

[B39] Millet D. Jacob D. J. Custer T. De Gouw J. Goldstein A. Karl T. (2008). New constraints on terrestrial and oceanic sources of atmospheric methanol. Atmos. Chem. Phys. 8, 6887–6905. 10.5194/acp-8-6887-2008

[B40] Monson R. K. Fall R. (1989). Isoprene emission from aspen leaves influence of environment and relation to photosynthesis and photorespiration. Plant Physiol. 90, 267–274. 10.1104/pp.90.1.267 16666747 PMC1061708

[B41] Nardini A. Pitt F. (1999). Drought resistance of *Quercus pubescens* as a function of root hydraulic conductance, xylem embolism and hydraulic architecture. New Phytol. 143, 485–493. 10.1046/j.1469-8137.1999.00476.x 33862892

[B42] Niinemets Ü. (2010a). Mild versus severe stress and BVOCs: thresholds, priming and consequences. Trends Plant Sci. 15, 145–153. 10.1016/j.tplants.2009.11.008 20006534

[B43] Niinemets Ü. (2010b). Responses of forest trees to single and multiple environmental stresses from seedlings to mature plants: past stress history, stress interactions, tolerance and acclimation. For. Ecol. Manage. 260, 1623–1639. 10.1016/j.foreco.2010.07.054

[B44] Niinemets Ü. Loreto F. Reichstein M. (2004). Physiological and physicochemical controls on foliar volatile organic compound emissions. Trends Plant Sci. 9, 180–186. 10.1016/j.tplants.2004.02.006 15063868

[B45] Oikawa P. Y. Lerdau M. T. (2013). Catabolism of volatile organic compounds influences plant survival. Trends Plant Sci. 18, 695–703. 10.1016/j.tplants.2013.08.011 24060580

[B46] Pazouki L. Niinemets Ü. (2016). Multi-substrate terpene synthases: their occurrence and physiological significance. Front. Plant Sci. 7:1019. 10.3389/fpls.2016.01019 27462341 PMC4940680

[B47] Pegoraro E. Rey A. Barron-Gafford G. Monson R. Malhi Y. Murthy R. (2005). The interacting effects of elevated atmospheric CO2 concentration, drought and leaf-to-air vapour pressure deficit on ecosystem isoprene fluxes. Oecologia 146, 120–129. 10.1007/s00442-005-0166-5 16001217

[B48] Peñuelas J. Staudt M. (2010). BVOCs and global change. Trends Plant Sci. 15, 133–144. 10.1016/j.tplants.2009.12.005 20097116

[B49] Pio C. Silva P. Cerqueira M. Nunes T. (2005). Diurnal and seasonal emissions of volatile organic compounds from cork oak (*Quercus suber*) trees. Atmos. Environ. 39, 1817–1827. 10.1016/j.atmosenv.2004.11.018

[B50] Polade S. D. Pierce D. W. Cayan D. R. Gershunov A. Dettinger M. D. (2014). The key role of dry days in changing regional climate and precipitation regimes. Sci. Rep. 4:4634. 10.1038/srep04364 24621567 PMC3952143

[B51] Pollastri S. Tsonev T. Loreto F. (2014). Isoprene improves photochemical efficiency and enhances heat dissipation in plants at physiological temperatures. J. Exp. Bot. 65, 1565–1570. 10.1093/jxb/eru033 24676032 PMC3967094

[B52] Pollmann J. Ortega J. Helmig D. (2005). Analysis of atmospheric sesquiterpenes: sampling losses and mitigation of ozone interferences. Environ. Sci. Technol. 39, 9620–9629. 10.1021/es050440w 16475343

[B53] Quézel P. Médail F. (2003). Ecologie et Biogéographie des Forêts du Bassin Méditerranéen. Paris: Elsevier.

[B54] Rodríguez-Calcerrada J. Buatois B. Chiche E. Shahin O. Staudt M. (2013). Leaf isoprene emission declines in *Quercus pubescens* seedlings experiencing drought–Any implication of soluble sugars and mitochondrial respiration? Environ. Exp. Bot. 85, 36–42. 10.1016/j.envexpbot.2012.08.001

[B55] Schade G. W. Goldstein A. H. (2006). Seasonal measurements of acetone and methanol: abundances and implications for atmospheric budgets. Glob. Biogeochem. Cycles 20:GB1011 10.1029/2005gb002566

[B56] Seco R. Penuelas J. Filella I. (2007). Short-chain oxygenated VOCs: emission and uptake by plants and atmospheric sources, sinks, and concentrations. Atmos. Environ. 41, 2477–2499. 10.1016/j.atmosenv.2006.11.029

[B57] Seco R. Penuelas J. Filella I. (2008). Formaldehyde emission and uptake by Mediterranean trees *Quercus ilex* and *Pinus halepensis*. Atmos. Environ. 42, 7907–7914. 10.1016/j.atmosenv.2008.07.006

[B58] Sharkey T. D. Yeh S. (2001). Isoprene emission from plants. Annu. Rev. Plant Biol. 52, 407–436. 10.1146/annurev.arplant.52.1.407 11337404

[B59] Simon V. Dumergues L. Bouchou P. Torres L. Lopez A. (2005). Isoprene emission rates and fluxes measured above a Mediterranean oak (*Quercus pubescens*) forest. Atmos. Res. 74, 49–63. 10.1016/j.atmosres.2004.04.005

[B60] Singh H. B. Salas L. J. Chatfield R. B. Czech E. Fried A. Walega J. (2004). Analysis of the atmospheric distribution, sources, and sinks of oxygenated volatile organic chemicals based on measurements over the Pacific during TRACE-P. J. Geophys. Res. 109, D15S07. 10.1029/2003JD003883

[B61] Smith M. D. Knapp A. K. Collins S. L. (2009). A framework for assessing ecosystem dynamics in response to chronic resource alterations induced by global change. Ecology 90, 3279–3289. 10.1890/08-1815.1 20120798

[B62] Tholl D. Boland W. Hansel A. Loreto F. Röse U. S. Schnitzler J. P. (2006). Practical approaches to plant volatile analysis. Plant J. 45, 540–560. 10.1111/j.1365-313X.2005.02612.x 16441348

[B63] Tie X. Guenther A. Holland E. (2003). Biogenic methanol and its impacts on tropospheric oxidants. Geophys. Res. Lett. 30, 1881 10.1029/2003GL017167

[B64] Velikova V. Várkonyi Z. Szabó M. Maslenkova L. Nogues I. Kovács L.. (2011). Increased thermostability of thylakoid membranes in isoprene-emitting leaves probed with three biophysical techniques. Plant Physiol. 157, 905–916. 10.1104/pp.111.182519 21807886 PMC3192565

[B65] Vickers C. E. Gershenzon J. Lerdau M. T. Loreto F. (2009). A unified mechanism of action for volatile isoprenoids in plant abiotic stress. Nat. Chem. Biol. 5, 283–291. 10.1038/nchembio.158 19377454

[B66] Vlasenko A. Macdonald A. Sjostedt S. Abbatt J. (2010). Formaldehyde measurements by Proton transfer reaction–Mass Spectrometry (PTR-MS): correction for humidity effects. Atmos. Measur. Tech. 3, 1055–1062. 10.5194/amt-3-1055-2010

[B67] von Caemmerer S. V. Farquhar G. (1981). Some relationships between the biochemistry of photosynthesis and the gas exchange of leaves. Planta 153, 376–387. 10.1007/BF00384257 24276943

