# Supplementary material for: Chronic Drought Decreases Anabolic and Catabolic BVOC Emissions of Quercus pubescens in a Mediterranean Forest
Source: Front Plant Sci. 2017 Feb 8;8:71. doi: 10.3389/fpls.2017.00071 (PMC5296328; doi:10.3389/fpls.2017.00071)
Supplement: Supplementary file 1 [file DataSheet1.PDF]

Chronic drought decreases anabolic and catabolic BVOC emissions of *Quercus pubescens* in a Mediterranean forest

Amélie Saunier[1*], Elena Ormeño[1], Henri Wortham[2], Brice Temime-Roussel[2], Caroline Lecareux[1], Christophe Boissard[3], Catherine Fernandez[1].

***Corresponding author**
amelie.saunier@imbe.fr

Figure S1: Cumulative precipitation for natural (ND) and amplified drought (AD) **A**) for 2012, **B**) for 20133 and **C**) for 2014.

Figure S2: Diurnal cycles for net photosynthesis (Pn) and stomatal conductance (Gw) according to season and drought treatment.

Figure S3: BVOCs emission rate of **A**) isoprene **B**) methanol **C**) isoprene oxidation products (MACR+MVK+ISOPOOH) **D**) methanol catabolic product (formaldehyde) according to natural (ND) and amplified drought (AD) over the seasonal cycle. Asterisks denote significant differences between treatments according to a two-way repeated measures ANOVA with (*) = $0.05 < P < 0.1$; * = $0.01 < P < 0.05$; ** = $0.001 < P < 0.01$ and letters denote significant differences among seasons according to Tukey *post hoc* test with a>b. Values are means ± S.E. and n=5.

Figure S4: Diurnal cycles of isoprene emission rates (left) and emission rates of isoprene oxidation products (MACR+MVK+ISOPOOH, right) averaged every hour. White points represent natural drought (ND) and black points represent amplified drought (AD). Values are means S.E. and n=5.

Figure S5: Diurnal cycles of methanol emission rates (left) and emission rates methanol oxidation product emission rate (formaldehyde, right) averaged every hour. White points represent natural drought (ND) and black points represent amplified drought (AD). Values are means S.E. and n=5.

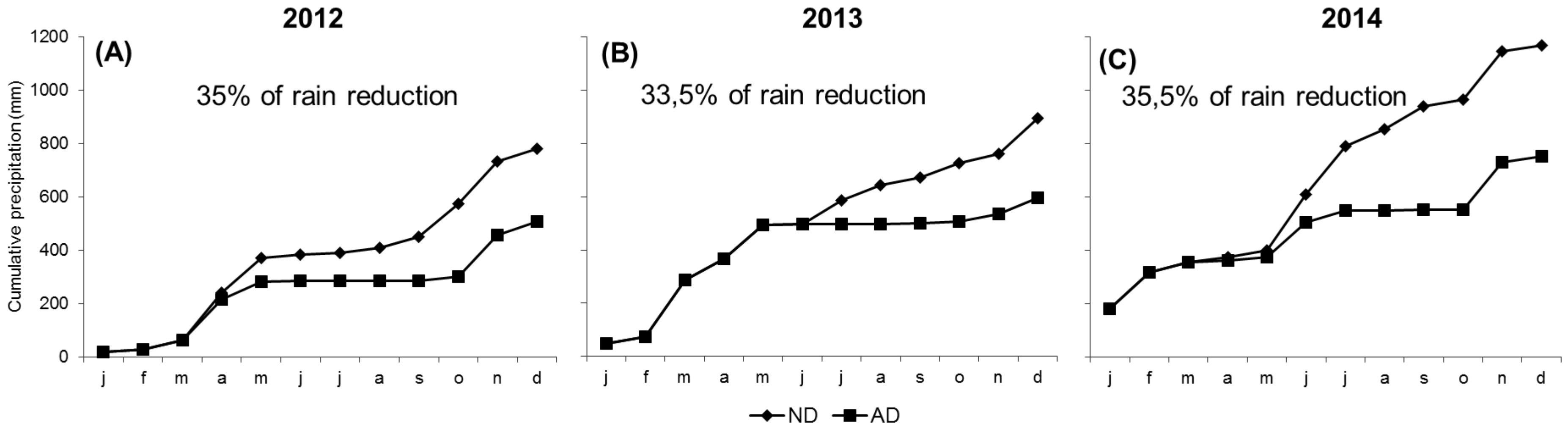

2012
2013
2014
(A)
(B)
(C)
35% of rain reduction
33,5% of rain reduction
35,5% of rain reduction
Cumulative precipitation (mm)
0
200
400
600
800
1000
1200
j f m a m j j a s o n d
ND
AD

Figure S1:

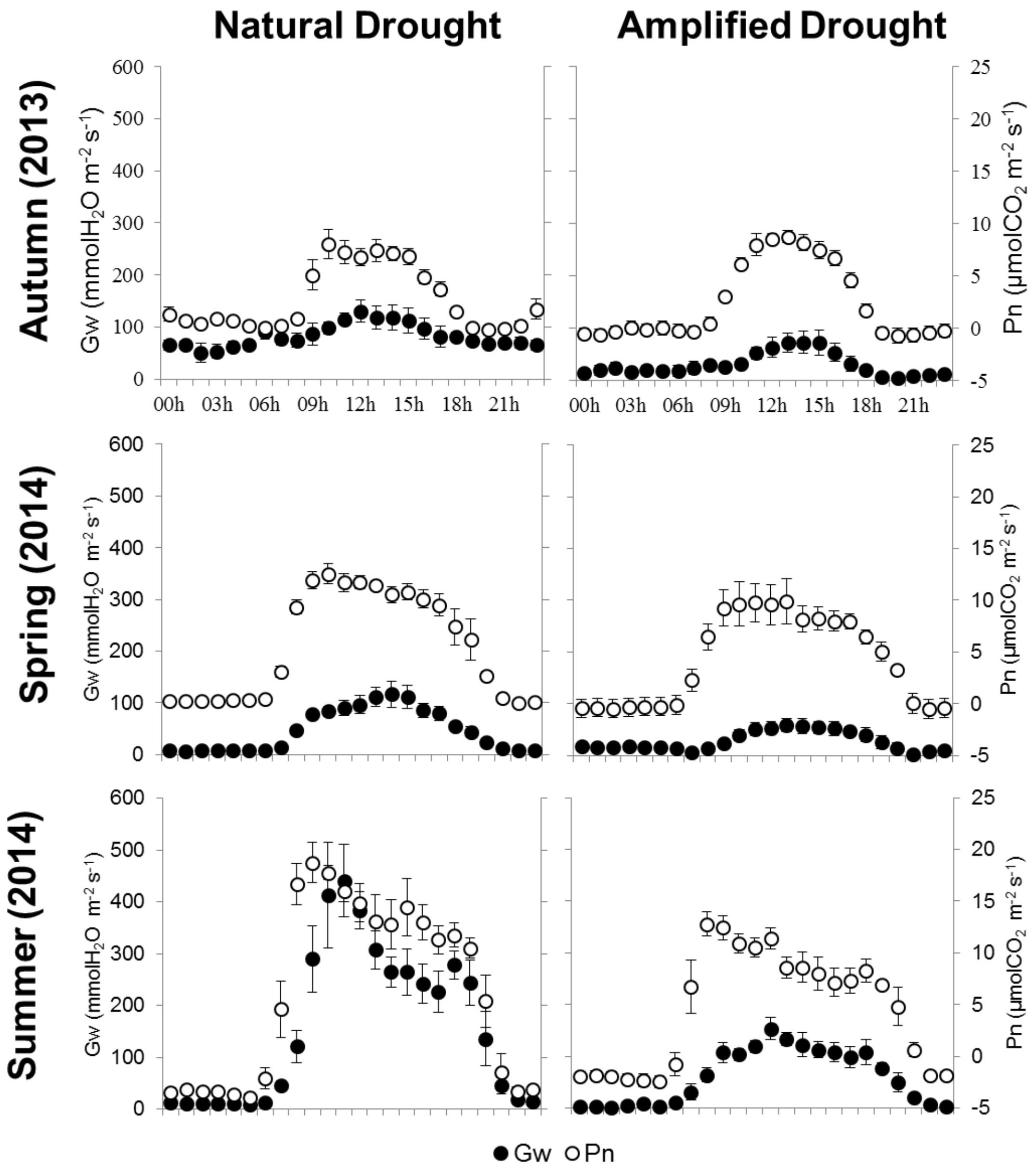

Natural Drought
Amplified Drought
Autumn (2013)
Spring (2014)
Summer (2014)
Gw (mmolH2O m-2 s-1)
Pn (µmolCO2 m-2 s-1)
00h 03h 06h 09h 12h 15h 18h 21h
Gw Pn

Figure S2:

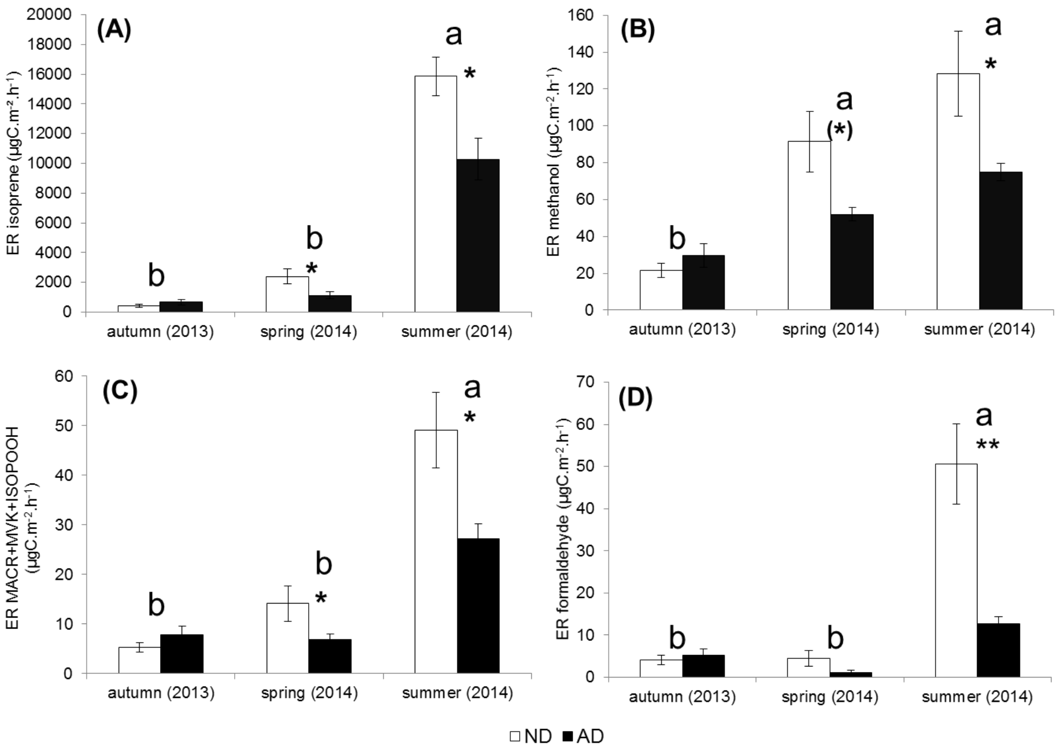
(A)
ER isoprene (µgC.m-2.h-1)
20000
18000
16000
14000
12000
10000
8000
6000
4000
2000
0
a
*
b
*
b
autumn (2013)
spring (2014)
summer (2014)
(B)
ER methanol (µgC.m-2.h-1)
160
140
120
100
80
60
40
20
0
a
*
a
(*)
b
autumn (2013)
spring (2014)
summer (2014)
(C)
ER MACR+MVK+ISOPOOH (µgC.m-2.h-1)
60
50
40
30
20
10
0
a
*
b
*
b
autumn (2013)
spring (2014)
summer (2014)
(D)
ER formaldehyde (µgC.m-2.h-1)
70
60
50
40
30
20
10
0
a
**
b
b
autumn (2013)
spring (2014)
summer (2014)
ND
AD

Figure S3:

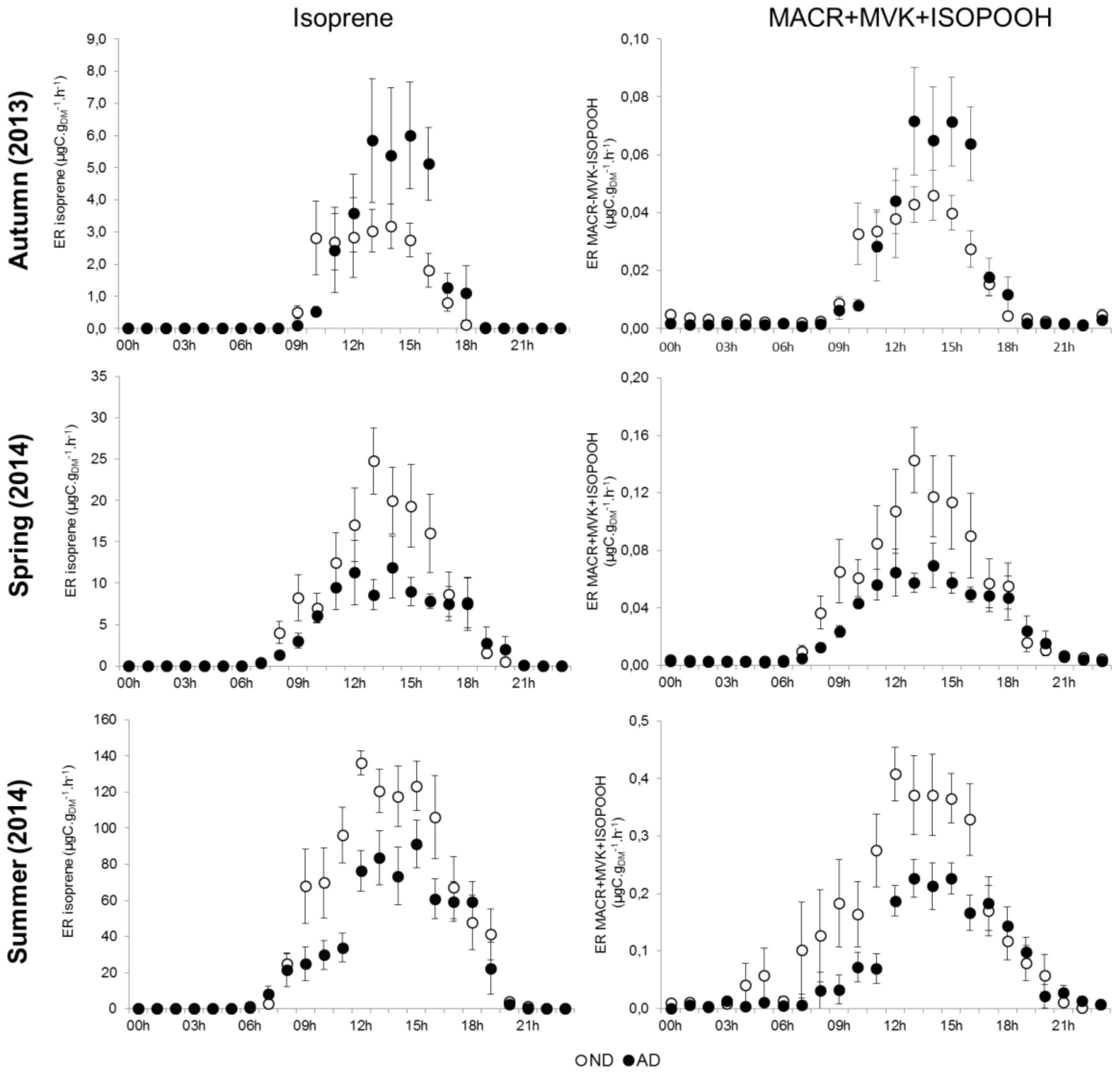

Isoprene
MACR+MVK+ISOPOOH
Autumn (2013)
Spring (2014)
Summer (2014)
ER isoprene (µgC.g_DM^-1.h^-1)
ER MACR-MVK-ISOPOOH (µgC.g_DM^-1.h^-1)
ER MACR+MVK+ISOPOOH (µgC.g_DM^-1.h^-1)
00h 03h 06h 09h 12h 15h 18h 21h
ND AD

Figure S4:

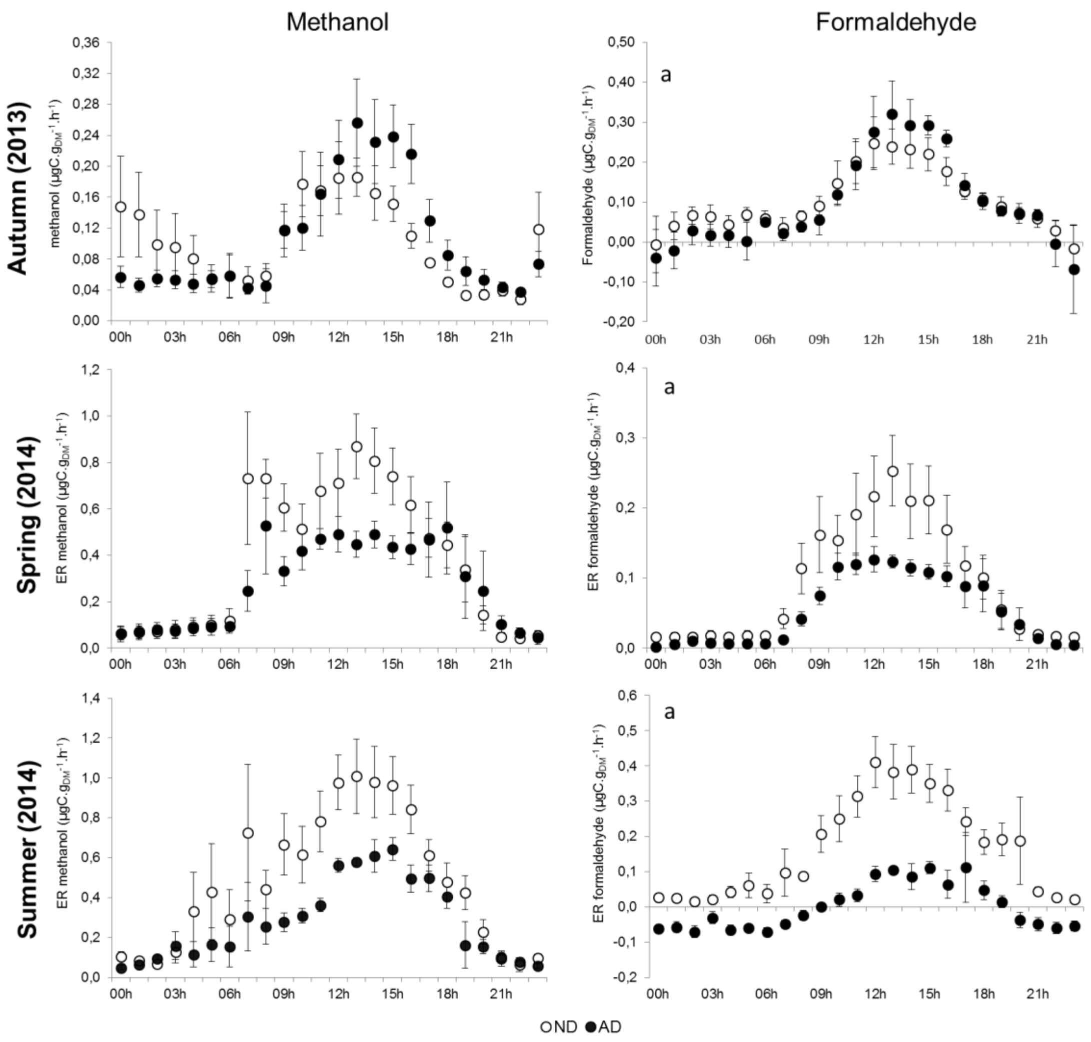
Methanol
Formaldehyde
Autumn (2013)
Spring (2014)
Summer (2014)
methanol (µgC.g$_{DM}^{-1}$.h^{-1})
Formaldehyde (µgC.g$_{DM}^{-1}$.h^{-1})
ER methanol (µgC.g$_{DM}^{-1}$.h^{-1})
ER formaldehyde (µgC.g$_{DM}^{-1}$.h^{-1})
a
00h 03h 06h 09h 12h 15h 18h 21h
ND AD

Figure S5:
